# Brugada Pattern Type 2 Diagnosis Unmasked by Aspiration Pneumonia

**DOI:** 10.7759/cureus.8331

**Published:** 2020-05-28

**Authors:** Yasar Sattar, Waqas Ullah, Syeda Ramsha Zaidi, Talal Almas, M. Chadi Alraies

**Affiliations:** 1 Internal Medicine, Icahn School of Medicine at Mount Sinai, New York City, USA; 2 Internal Medicine, Abington Hospital-Jefferson Health, Abington, USA; 3 Internal Medicine, St. Mary Mercy Hospital, Livonia, USA; 4 Internal Medicine, Royal College of Surgeons in Ireland, Dublin, IRL; 5 Cardiology, Detroit Medical Center, Detroit, USA

**Keywords:** brugada syndrome, brugada pattern, aspiration pneumonia

## Abstract

Brugada syndrome (BrS) is a rare autosomal dominant mutation affecting sodium channels. Electrocardiography can show two Brugada patterns (BrP). Type 1 BrP usually causes sudden cardiac arrest (SCA). Type 2 BrP can appear during circumstances that result in delayed sodium channel opening, such as fever, pneumonia, or use of sodium channel blockers. Patients with type 2 BrP often have underlying type 1 BrP; this can be confirmed by an ajmaline challenge test. We describe the case of a patient who presented with SCA. He later had an interval type 2 BrP secondary to aspiration pneumonia, followed by type 1 BrP pattern confirmed by an ajmaline challenge test. The patient ultimately underwent implantable cardiac defibrillator placement to prevent future SCA.

## Introduction

Brugada syndrome (BrS) was initially described by Pedro and Joseph Brugada in 1992 [1]. BrS is an autosomal dominant mutation affecting sodium channels, which can provoke sudden cardiac arrest (SCA) owing to ventricular arrhythmia [1,2]. Electrocardiographic (ECG) characteristics of BrS can follow one of two Brugada patterns (BrP): the type 1 pattern features a coved-type ST segment, while the type 2 BrP features a saddleback ST segment in precordial leads V1-V3 [3]. Patients with BrS can present with a normal initial ECG; however, BrP can be unmasked by specific stressors, such as infection, fever, psychosis, medications including sodium channel blockers, propranolol, tricyclic antidepressants, and illicit drugs such as cocaine [4]. We describe a case of a patient with BrS who presented with SCA, followed by an interval appearance of type 2 BrP in the setting of aspiration pneumonia.

## Case presentation

**Figure 1:**
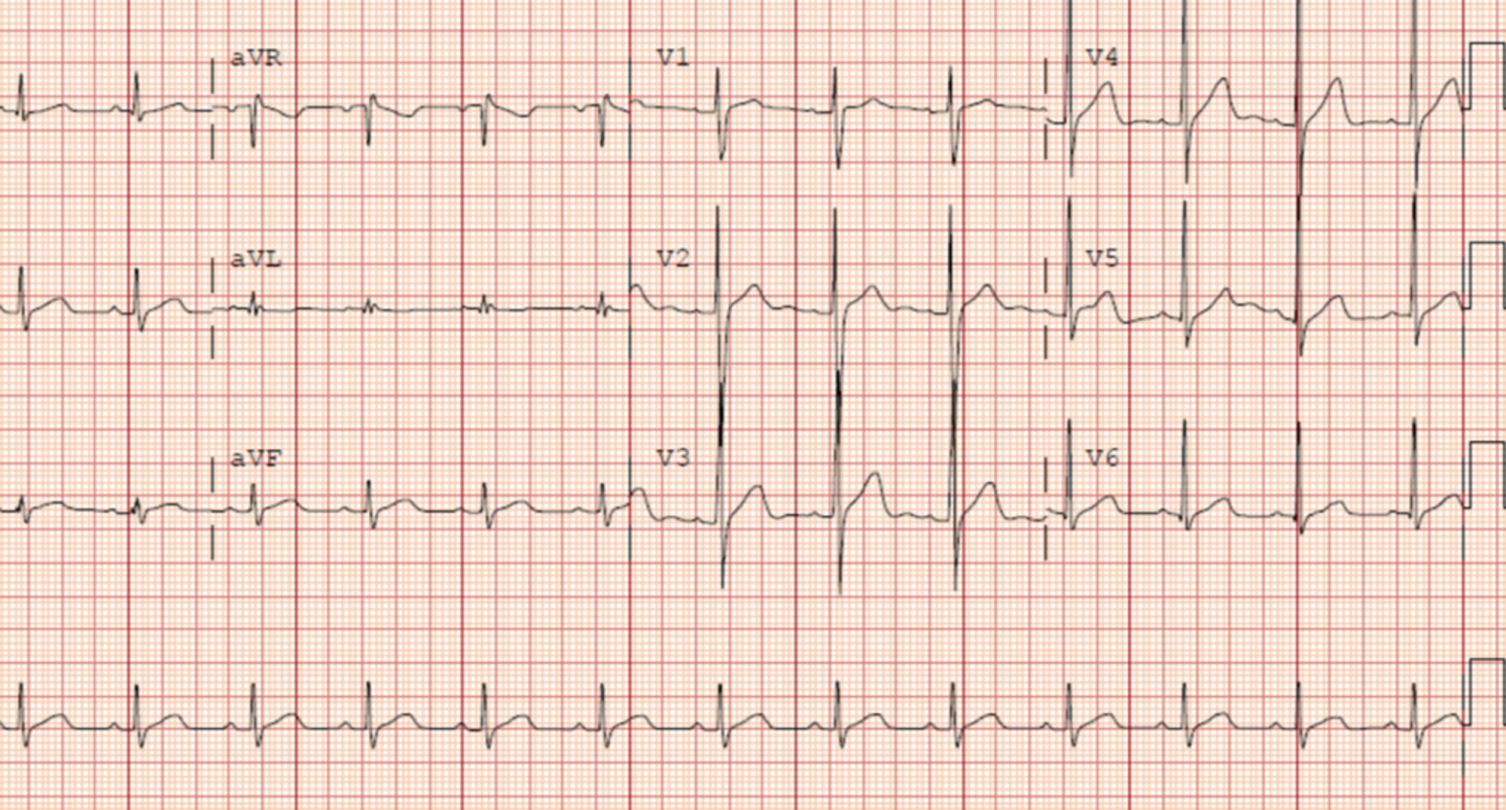
Electrocardiogram revealing normal sinus rhythm, no signs of ischemia, and normal intervals.

A 21-year-old man with no significant past medical history was brought to the emergency department (ED) by emergency medical services for SCA with ventricular fibrillation (VF) in the field. He was ultimately shocked four times and given two rounds of epinephrine prior to the return of spontaneous circulation. Upon arrival at the ED, he had regained pulses and was started on minimal vasopressors and low tidal volume minute ventilation (LTVMV). His initial vital signs and breathing status were stable. An initial electrocardiogram (ECG) was performed, which revealed a sinus rhythm (Figure 1).

**Figure 2:**
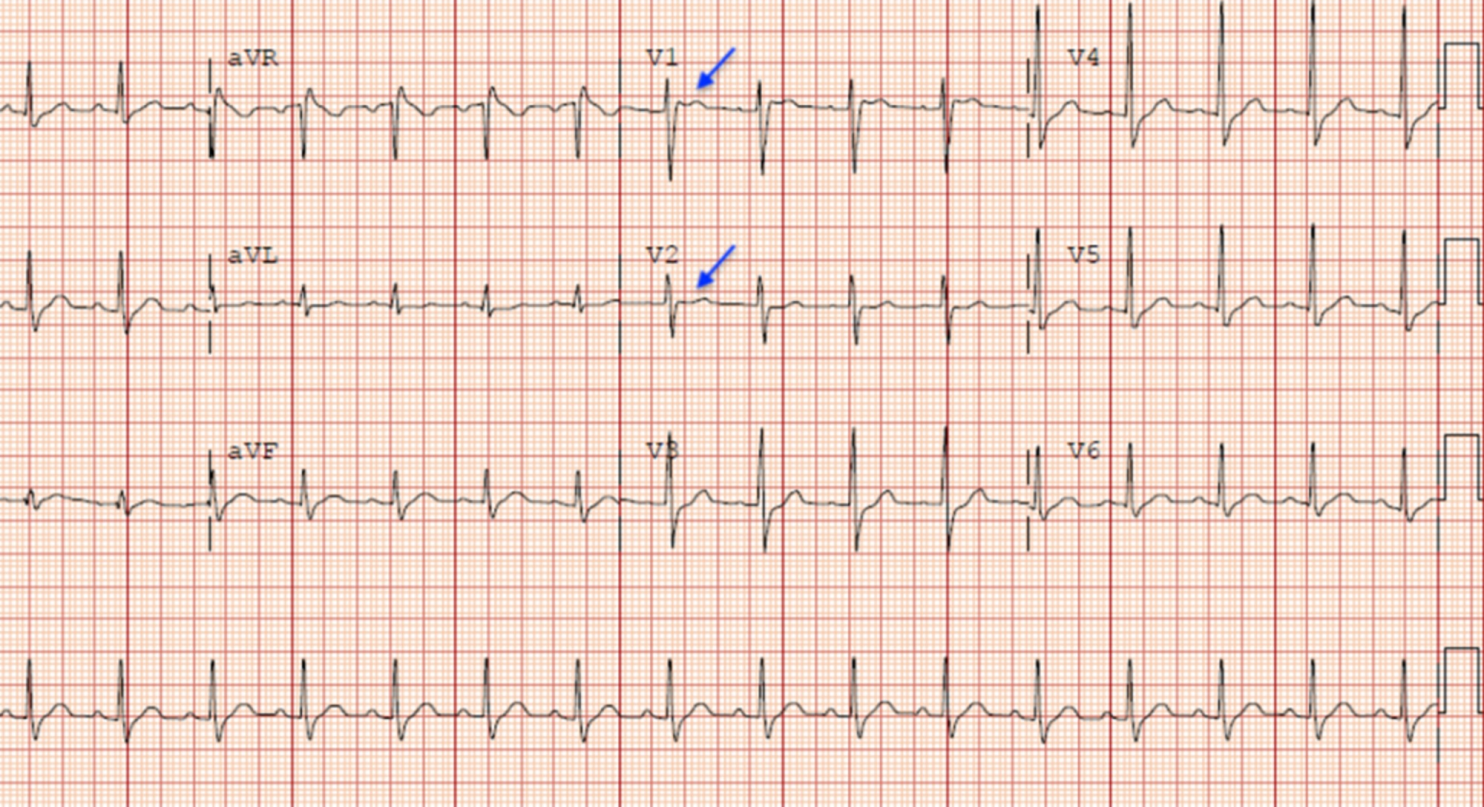
Electrocardiogram revealing type 2 Brugada pattern showing saddleback ST pattern in V1-V2 (arrows).

The findings of his physical examination were non-significant; he had intact brainstem reflexes and normal cardiopulmonary exam on ventilation. After one day in the intensive care unit (ICU), his vital signs were as follows: temperature, 101°F; blood pressure, 110/70 mmHg; heart rate, 90 beats/minute; respiratory rate, 22 breaths/minute; and oxygen saturation, 98% on LTVMV. A repeat ECG during an interval fever episode revealed a saddleback T wave in precordial leads V1-V2 consistent with type 2 BrP (Figure 2).

**Figure 3:**
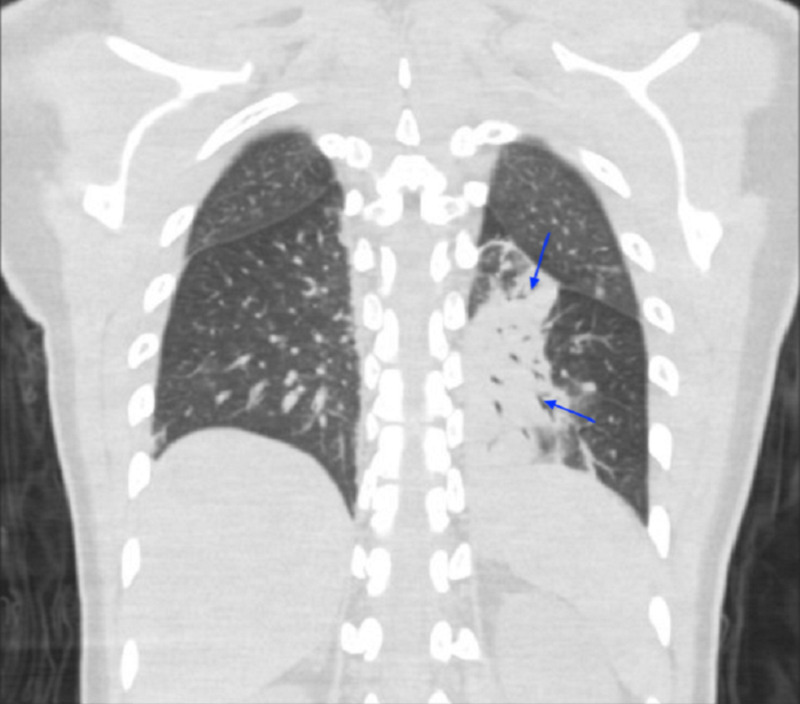
CT of the chest (coronal plane) showing infiltrates in the mid and lower left lung fields.

CT of the chest showed patchy bilateral infiltrates consistent with aspiration pneumonia (Figure 3).

Initial transthoracic echocardiography showed diffusely hypokinetic wall motion with a left ventricular ejection fraction (LVEF) of 20%; improvement to normal LVEF was noted within 24 hours (Video 1).

**Video 1:** Normal four-chamber transthoracic echocardiography showing normal chambers and valve motion.

Because of the patient’s SCA and ECG findings, he was presumed to have type 2 BrP provoked by fever and pneumonia. The patient was initially started on low-dose norepinephrine, fluids, vancomycin/cefepime, and LTVMV, with successful weaning.

The patient was monitored via follow-up in the cardiology clinic, where he tested positive to an ajmaline challenge test for type 1 BrP. The patient underwent implantable cardiac defibrillator (ICD) placement during outpatient follow-up.

## Discussion

Pathophysiology

BrS is an autosomal mutation affecting sodium channels. The genetic mutation in BrS commonly affects epicardial and M cells of the heart. Some of the common mutations involve the genes SCN10A and SCN5A [5]. Mutations in sodium channels lead to a decrease in the inward current of sodium, causing shorter action potentials [6]. The shortening of action potentials due to mutations is further enhanced by an increased counter outward current of sodium, as found in the right ventricular outflow tract in some patients. This causes reduced initial depolarization and delayed phase 2 due to reduced co-activation of calcium channels. These voltage changes of sodium channels appear as ST-segment elevation and T-wave inversion seen in the right precordial leads [7]. The effect of mutated sodium channels can be further enhanced by risk factors, such as fever, psychosis, medications including sodium channel blockers, propranolol, tricyclic antidepressants, or illicit drugs like cocaine.

Clinical presentation and diagnostic criteria

While the clinical manifestations of BrS may vary, BrP is usually asymptomatic. The presence of a type 1 BrP subtype portends to a worse prognosis and predisposes to debilitating clinical outcomes such as SCA. Clinical presentation of BrS can also include atrial tachyarrhythmias and life-threatening ventricular arrhythmias, including VF and ventricular tachycardia (VT). Syncope or SCA can be one of the sequelae of tachyarrhythmias, and establishing a diagnosis of BrS mainly involves clinical symptoms and ECG findings.

In 2005, the Heart Rhythm Society (HRS) and the European Heart Rhythm Association (EHRA) stipulated specific criteria for BrP types 1 and 2 [8]. The HRS and EHRA stated that type 1 BrP includes coved-type ST-segment elevations in precordial leads (V1-V2) with or without the use of sodium channel blockers [9]. The criteria for type 2 BrS included a saddleback-type ST-segment elevation in more than one precordial lead (V1-V2) at baseline but conversion to coved type in similar precordial leads upon administration of sodium channel blockers [9]. To meet the requisite criteria, patients must also demonstrate at least one of the following: family history of type 1 BrP/BrS, VF, VT, or nocturnal agonal respiration [9]. Initial ECG findings are often unremarkable in BrP and BrS, and risk factor such as infections, fever, psychosis, medications (e.g., sodium channel blockers, propranolol, tricyclic antidepressants), and illicit drugs (e.g., cocaine) should prompt more frequent ECG workup for BrS and BrP [3].

BrP can be divided into three subtypes, each varying with pertinence to the typical ECG findings that they elicit. It is imperative to note, however, that findings compatible with BrP type 1 are necessary to yield a diagnosis of BrS, owing primarily to the fact that ECG findings inherent to types 2 and 3 are also commonly found in patients who have no indication of a BrS [10]. Thus, while type 2 and 3 findings can insinuate a diagnosis of BrS, challenge testing is necessary for establishing and confirming the diagnosis of BrS. While the type 1 pattern is usually associated with cases of SCA, it can often masquerade as BrP type 2 upon workup [11]. Interestingly, the eventual diagnosis of a BrP type 1 is usually clinically divulged in the aftermath of concomitant infection, such as pneumonia. Similarly, in our case, our patient initially presented with a typical type 2 BrP but was later also found to be positive for type 1 in the wake of the ajmaline (sodium blocker) challenge test.

Treatment

Treatment for BrS is centered around the prevention of ventricular arrhythmias and SCA through the means of an ICD. An ICD is indicated in patients with documented electrophysiological ailments, survivors of SCA, and patients with a family history of SCA. Patients with BrP do not require any specific therapy in the absence of strong family history and an electrophysiology study provoking ventricular arrhythmias. ICD is extremely safe and highly efficacious in terminating ventricular arrhythmias in patients with SCA. It is, therefore, an essential component of the treatment regimen [12].

## Conclusions

BrS can initially present as type 2 BrP with SCA. Initial ECG findings can be unremarkable, and frequent ECGs should be performed during exposure to exacerbating risk factors such as pneumonia, as these circumstances can unmask the pathognomonic findings of a type 2 BrP. Confirmatory test with ajmaline challenge should be done for BrP type 2 to find any type 1 pattern, as this would increase the risk of future SCA. Treatment with ICD placement is preferred to prevent future SCA in patients with diagnosed BrS.
